# Publisher Correction: Reconciling qualitative, abstract, and scalable modeling of biological networks

**DOI:** 10.1038/s41467-020-18766-1

**Published:** 2020-09-24

**Authors:** Loïc Paulevé, Juri Kolčák, Thomas Chatain, Stefan Haar

**Affiliations:** 1 Université Bordeaux, Bordeaux INP, CNRS, LaBRI, UMR5800, 351 cours de la Libération, Talence, 33400 France; 2 LRI UMR8623, Université Paris-Sud, CNRS, Université Paris-Saclay, Bat 650 Ada Lovelace, Rue Raimond Castaing, Gif-sur-Yvette, 91190 France; 3 Inria and LSV, CNRS (UMR 8643) and ENS Paris-Saclay, Université Paris-Saclay, 4 avenue des Sciences, Gif-sur-Yvette, 91190 France

**Keywords:** Computer modelling, Computer science, Dynamic networks, Dynamical systems, Regulatory networks

Correction to: *Nature Communications* 10.1038/s41467-020-18112-5, published online 26 August 2020.

In the original version of this Article errors were introduced into Figs. 1 and 2 during the typesetting process. In Fig. 1b the logic of activities read:

f_1_ (**x**) = signal

f_1_ (**x**) = **x**_**1**_

f_1_ (**x**) = not **x**_**1**_ and **x**_**2**_

It should read:

f_1_ (**x**) = signal

f_2_ (**x**) = **x**_**1**_

f_3_ (**x**) = not **x**_**1**_ and **x**_**2**_

In Figure 2b the Boolean network read:

f_1_ (**x**) = not **x**_**2**_

f_1_ (**x**) = not **x**_**1**_

f_3_ (**x**) = not **x**_**1**_ and **x**_**2**_

It should read:

f_1_ (**x**) = not **x**_**2**_

f_2_ (**x**) = not **x**_**1**_

f_3_ (**x**) = not **x**_**1**_ and **x**_**2**_

These errors have been fixed in the HTML and PDF versions of the Article.

